# The Fatty Acid Lipid Metabolism Nexus in COVID-19

**DOI:** 10.3390/v13010090

**Published:** 2021-01-11

**Authors:** Jerome E. Tanner, Caroline Alfieri

**Affiliations:** 1 Laboratory of Viral Pathogenesis, Research Centre, CHU Sainte-Justine, 3175 Côte Sainte-Catherine Road, Montréal, QC H3T 1C5, Canada; jerome.tanner.hsj@ssss.gouv.qc.ca; 2 Département de Microbiologie, Infectiologie et Immunologie, Université de Montréal, 2900 Boul. Édouard-Montpetit, Montréal, QC H3T 1J4, Canada

**Keywords:** endoplasmic reticulum stress response, unfolded protein response, mTORC1, SREBP-1, nonstructural protein, membrane protein, spike protein, envelope protein, replicative organelle, antiviral

## Abstract

Enteric symptomology seen in early-stage severe acute respiratory syndrome (SARS)-2003 and COVID-19 is evidence of virus replication occurring in the intestine, liver and pancreas. Aberrant lipid metabolism in morbidly obese individuals adversely affects the COVID-19 immune response and increases disease severity. Such observations are in line with the importance of lipid metabolism in COVID-19, and point to the gut as a site for intervention as well as a therapeutic target in treating the disease. Formation of complex lipid membranes and palmitoylation of coronavirus proteins are essential during viral replication and assembly. Inhibition of fatty acid synthase (FASN) and restoration of lipid catabolism by activation of AMP-activated protein kinase (AMPK) impede replication of coronaviruses closely related to SARS-coronavirus-2 (CoV-2). In vitro findings and clinical data reveal that the FASN inhibitor, orlistat, and the AMPK activator, metformin, may inhibit coronavirus replication and reduce systemic inflammation to restore immune homeostasis. Such observations, along with the known mechanisms of action for these types of drugs, suggest that targeting fatty acid lipid metabolism could directly inhibit virus replication while positively impacting the patient’s response to COVID-19.

## 1. Introduction

All viruses repurpose cell catabolism and anabolism to generate energy and macromolecules for efficient replication; however, the mechanisms and consequences of SARS-coronavirus-2 (CoV-2) lipid metabolic reprogramming is largely unexplored. In this review, we examine fatty acid lipid metabolism in the context of COVID-19 at the organismal, cellular and macromolecular levels. With this basis of understanding, we propose two rational treatment options that directly target the virus’s lipid dependency as well as strengthen the patient’s response to SARS-CoV-2 infection.

## 2. Digestive System Involvement in COVID-19

Mammalian cell proliferation and the formation and maintenance of their larger organ system counterparts require an adequate supply of energy and cellular building blocks including fatty acid phospholipids which represent the major constituent of biological membranes [1]. Changes in membrane phospholipid composition and acyl length determine the biophysical properties of cell membranes which in turn impact larger biological processes. Mammalian lipid absorption, synthesis and composition for maintaining systemic homeostasis are controlled mainly in the intestine, liver and adipose tissues [2]. As exemplified by the hepatitis C virus (HCV), enveloped RNA viruses can alter lipid homeostasis to enhance virus replication and increase infectivity [3]. Early presentation of gastrointestinal symptoms recently seen in COVID-19 patients was common in SARS-2003 patients; these often include diarrhea, nausea, vomiting and abdominal pain [4,5,6,7,8]. Further, viable virus was detectable in patient stool samples and in sewage, which can contribute to community spread [6,9,10,11,12]. As noted by the increase in serum alanine aminotransferase, aspartate transaminase and glycemic index, SARS-CoV patients develop mild to severe liver or pancreatic dysfunction as the disease progresses [13,14,15]. The extent of liver and intestinal damage was noted upon examination of biopsies from fatal SARS-2003 cases [16]. Liver and enteric tissue showed fatty degeneration, cloudy swelling, apoptosis and dot necrosis of hepatocytes, as well as regional hemorrhage and vascular congestion with lymphocytic infiltration in gastrointestinal wall tissue and in hepatic and pancreatic ducts. Transient or permanent pancreatic dysfunction seen in SARS-CoV infection [15] is the result of β-cell damage and likely stems from β-cell inflammokine activation of autoreactive T cells and macrophages and, if extensive, can lead to fulminant and permanent diabetes [17]. These disparate SARS-CoV digestive organ pathologies all stem from the fact that intestinal, hepatic and pancreatic cells express one or more of the coronavirus receptors, namely angiotensin-converting enzyme-2 (ACE2), dipeptidyl peptidase-4 (CD26) and CD209L [18,19,20]. The commonality of intestine, liver and pancreatic β-cell involvement noted in SARS-2003 and COVID-19 points to the digestive tract as a significant site of disease with the potential to alter lipid metabolism.

## 3. Potential Impact of Elevated Systemic and Cellular Fatty Acid Levels in Obese Individuals Infected with SARS-CoV-2 

The number of COVID-19 patients requiring hospitalization is three-fold greater for individuals who are morbidly obese (BMI > 40) [21]. When present as a comorbidity with type 2 diabetes mellitus (T2DM), obesity increases patient risk of death ten-fold [14]. 

Numerous metabolic disturbances resulting from obesity contribute to a chronic state of low-grade inflammation and a diminished host response to viral infection [22,23]. Elevated serum triacylglycerol (TAG), free fatty acids (FFA), and proinflammatory adipokines leptin and resistin, along with TNF-α, IL-6, IL-1β, IL-18 and MCP-1 concurrent with lower amounts of the anti-inflammatory adipokine, adiponectin, collectively weaken the innate and adaptive immune system [24]. Following infection of an obese individual, type I interferon levels are reduced, and the natural killer cell response is attenuated. This dampened response initiates a cycle of decreased IL-12, IL-18, IFNγ and IL-2 responses, together with lowering of the adaptive immune response [22,23]. Long-term obesity also promotes chronic inflammation and a loss of gut mucosal integrity [25,26]. One can speculate that compromised immune function coupled with weakened gut integrity, precipitated by obesity, provides SARS-CoV a greater opportunity to establish primary enteric infection and better ease of access to circulatory spread. 

At the cellular level, accumulation of excess fatty acid stemming from obesity causes chronic endoplasmic reticulum (ER) stress response and continual activation of the unfolded protein response (UPR) pathway [27,28]. Under normal conditions, the ER provides the cellular machinery needed for proper protein folding, maturation, and directed trafficking of glycosylated and secretory proteins. The ER is also needed for calcium homeostasis and metabolism of complex lipids. An imbalance in these ER demands activates the evolutionarily conserved UPR pathway in an attempt to stem protein production, enhance removal of misfolded protein and control the synthesis of cellular lipid [29]. When the ER protein-folding capacity is exceeded and dysfunctional proteins accumulate, activation of the UPR pathway attempts to restore ER homeostasis by temporarily reducing global protein synthesis, enlarging ER volumes through endomembrane restructuring, increasing ER-folding capacity through the up-regulation of chaperones and foldases, and increasing protein turnover capacity through the up-regulation of ER-associated degradation (ERAD) components and ER-specific autophagy [30,31]. If ER homeostasis is unattainable, UPR initiates cell-death programs to eliminate the defective cell for the benefit of the organism [32]. The ‘restore or die’ decision by the cell is controlled and directed by the inositol-requiring enzyme 1 (IRE1), the activating transcription factor 6 (ATF6) and the protein kinase RNA (PKR)-like ER kinase or PERK. At times of severe ER stress and failure to restore ER homeostasis, the UPR network is forced to engage in additional SOS mechanisms, including ER-associated degradation (ERAD), autophagy or even cell death by apoptosis. In obese individuals, where ER stress is chronic and UPR pathways are already heightened, the added stress on the ER caused by SARS-CoV-2 replication could easily shift UPR signaling beyond homeostasis restoration towards apoptosis and greater tissue destruction [33]. 

## 4. The Essential Role of Lipids during SARS-CoV-2 Replication and Virion Assembly

SARS-CoV-2 replication relies on newly synthesized phospholipids or reengineered host membrane vesicles to serve as the replicative organelle that coordinates pairing of viral genomes and protein synthesis with virus assembly to complete the virus replication cycle [34,35,36,37]. Coronavirus replicative organelles are comprised of 200 nm-wide double-membrane vesicles (DMV) derived from the ER-Golgi vesicular transport system [38,39]. It is hypothesized that DMV structures more efficiently concentrate newly synthesized viral RNA genomes with associated virion structural proteins to better enable virus assembly and packaging. DMV could also serve to shield viral genome RNAs from recognition and destruction by innate cellular defense systems [40,41]. In concert with host cell ER factors and an increase in fatty acid synthesis [37], the SARS-CoV-2 nonstructural proteins (nsp)3, 4, 6 construct the replication organelle structures [42,43]. Nsp3 was shown to initiate the formation of large multilamellar vesicles which are then refined with the aid of nsp4 into extensive DMV pairings and maze-like bodies. Nsp6 contributes to replication organelle formation by potentially promoting cellular autophagy and membrane lipid recycling [44]. Once completed, replication organelles act as scaffolds for the coronavirus membrane protein that in turn coordinates virus RNA-nucleocapsid assembly and subsequent lipid envelopment of virion spike and envelope proteins. Assembled coronavirus particles bud into the ER-Golgi vasculature for spike and envelope glycosylation and subsequent virion egress through the cell’s secretory pathway and outside the cell [45].

Beyond providing a safe place for SARS-CoV-2 assembly, ER stress and activation of the UPR pathway incurred during virus-induced organelle remodeling can be gainfully exploited by the virus. ER UPR activation stimulates global lipid synthesis. UPR activation of IRE1 signaling results in proper RNA splicing and translation of X-box binding protein 1 (XBP1) mRNA which in turn stimulates sterol regulatory-element binding protein-1 (SREBP-1) and transcription of anabolic lipid genes including fatty acid synthase (FASN), acetyl-CoA carboxylase (ACC) and stearoyl-CoA desaturase 1 (SCD1) [46]. Thus UPR activation caused by SARS-CoV can provide much needed lipid stocks during virus and replicative organelle assembly [46]. As an added virus benefit, increased lipid stocks allow for expanded DMV luminal volumes resulting in a decreased effective concentration of misfolded virus proteins and preventing the possibility of UPR-induced apoptosis [29]. 

## 5. The Essential Role of Lipid Addition to SARS-CoV Proteins

**Figure 1:**
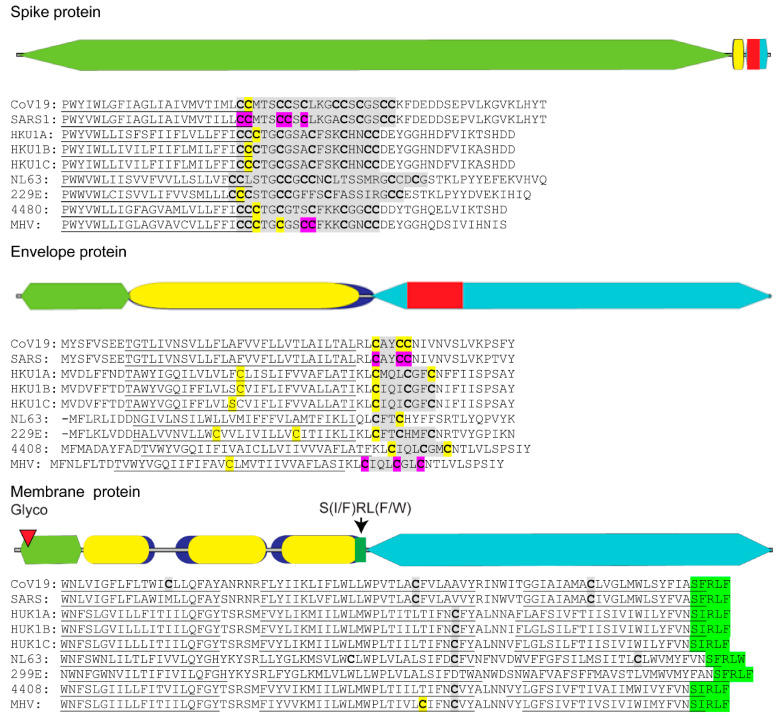
Distribution of palmitoylated cysteine residues in coronavirus structural proteins. Diagram of SARS-CoV-2 spike, envelope and membrane proteins depicting the relative amino acid length of their ectodomain (olive green), hydrophobic domain (blue) with internal transmembrane sequences (mustard), endodomain (teal), *N*-glycosylation (maroon triangle), conserved S(I/F)RL(F/W) motif (bright green) and cysteine clusters of proven palmitoylation (red). Actual sequences of cysteine clusters (grey highlight) with individual cysteine residues (bold) found in or near predicted transmembrane domains (underlined) for human and mouse coronavirus spike, envelope and membrane proteins are shown. In silico predicted individual cysteines (yellow) and biochemically confirmed (purple) palmitoylation sites are shown. Coronavirus spike, envelope and membrane protein amino acid sequences were derived from GenBank accession IDs for SARS-CoV-2, NC_045512; SARS-CoV-1, AY291315; human coronavirus HUK1 genotype A, AY597011; HUK1 genotype B, AY884001; HUK1 genotype C, DQ415898; human coronavirus NL63, NC_005831; 299E, AF304460; human enteric coronavirus strain 4408, FJ938067; and related murine hepatitis virus strain A59 MHV, MF618252. Coronavirus protein alignments with hydrophobic transmembrane locations were performed using Clustal Omega and TMHMM v. 2.0 programs, respectively. Identification of potential palmitoylation targets was performed using CSS-lm 4.0 and Palm-GPS algorithms set for high stringency.

SARS-CoV requires addition of lipid chains to conserved cysteine residues located adjacent to the transmembrane sections of the virus spike and envelope proteins (Figure 1) [47,48,49]. Lipid addition occurs through the process of cysteine palmitoylation [50]. Cysteine palmitoylation reversibly adds palmitate (C16:0), or the less common stearate (C18:0) or arachidonate (C20:0) moieties to cysteine residues that dynamically increase a protein’s affinity for cellular membranes and hydrophobic pockets on neighboring proteins or protein domains. Beyond serving as a membrane tether, protein palmitoylation promotes protein shuttling between different membrane compartments [51]. Palmitoylated proteins are often categorized by their site of lipid addition and include ones with palmitoylated cysteines lying close to (≤20 amino acid distance) or within a given protein’s transmembrane spanning sequence, located at the carboxyl-terminus or near the amino-terminus, and expressing the MGC motif [52]. 

Palmitoylation of viral envelope proteins usually occurs on cysteine residues located within or near their transmembrane domain [50]. Cysteine palmitoylation is catalyzed by the DHHC (Asp-His-His-Cys) rich domain palmitoyl acyltransferases that reside in the ER and Golgi [53]. These integral membrane proteins transfer palmitate residing in the DHHC motif to an unreduced cysteine target residue in the acceptor protein. Of the three coronavirus virion membrane proteins, namely spike, envelope and membrane, only the spike and envelope proteins were shown to be palmitoylated and important in virus replication [54,55]. Spike and envelope palmitoylation was shown to increase protein trafficking to replication organelles and aid in assembly of the virus envelope. Detailed examination of the SARS-CoV-2 spike protein endodomain reveals that it can be divided into a cysteine-rich cluster located near the viral envelope and a carboxy terminal region containing both basic and acidic residues (Figure 1). Palmitoylation of specific cysteine residues in the spike protein cysteine cluster for the related SARS-CoV-1 and mouse hepatitis virus (MHV) was found to be essential for efficient virus assembly and during initial infection when the spike protein-host cell membrane fusion event occurred (Figure 1, purple) [49,55]. It has been hypothesized that spike protein palmitoylation adds additional anchoring properties to the spike protein fusion domain allowing for optimum host cell fusion and virus entry by lessening torsional strain in the spike protein transmembrane domain [55]. 

Coronavirus envelope protein is also palmitoylated (Figure 1) [54,56]. Although abundantly expressed in the ER of infected cells, only a small portion of the envelope is incorporated into the virion structure [57]. Close association of the coronavirus envelope protein with cell membranes and other viral envelope proteins induces ER membrane curvature and allows nucleocapsid envelopment during virus budding into ER-Golgi vasculature [58]. A second property of the envelope protein is the formation of a homo-pentamer calcium ion channel expected to relieve ER stress by maintenance of calcium homeostasis, thus preventing UPR-induced apoptosis [59,60]. When occurring in close proximity to the envelope protein transmembrane sequence, palmitoylation may serve to strengthen tethering of envelope protein to the DMV. 

**Figure 2:**
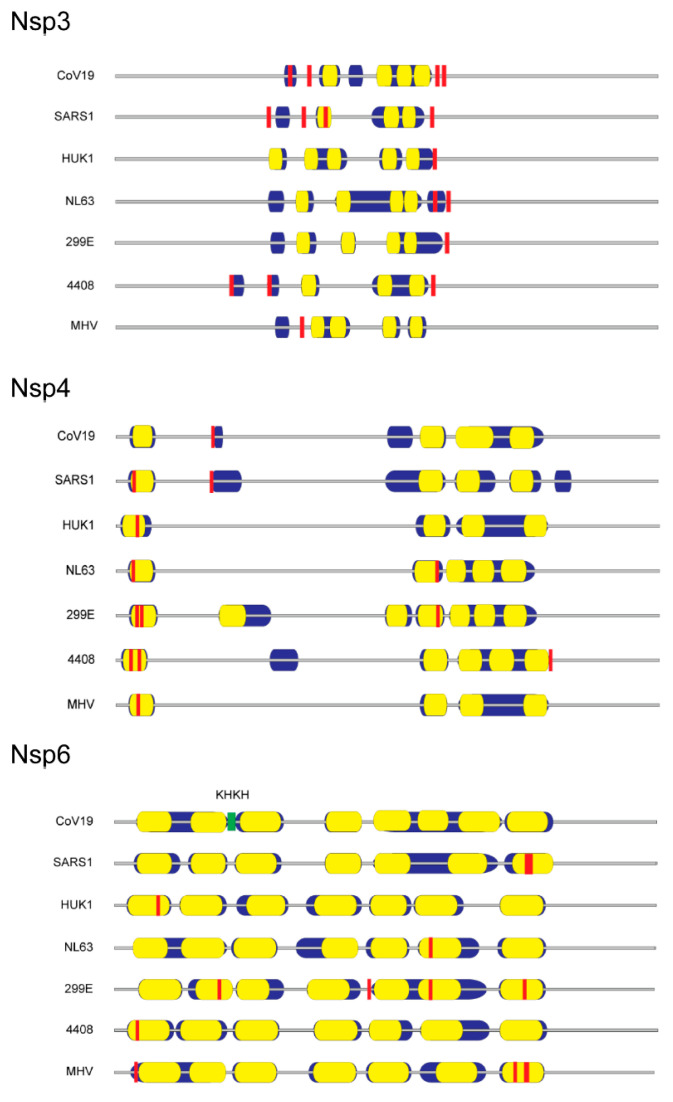
Distribution of potential membrane-associated palmitoylated cysteine residues in coronavirus nonstructural proteins 3, 4 and 6. Diagrams show relative amino acid length with predicted hydrophobic domains (blue), internal transmembrane sequences (yellow), and consensus sites of cysteine palmitoylation (red). Coronavirus nonstructural protein sequences were obtained from GenBank accession IDs listed in Figure 1.

Published evidence is lacking with respect to cysteine palmitoylation of other SARS-CoV-2 proteins intimately involved with lipid membranes, namely the virion membrane protein and nsp3, 4 and 6. We postulate that if cysteine palmitoylation of these proteins were essential to their function, including tethering to the viral lipid envelope or to the DMV, the cysteine residue would be conserved among other β-coronaviruses and be located close to or within their transmembrane sequence(s). In silico alignments of human coronaviruses, including SARS-CoV, with the prototypic MHV membrane protein or nsp3, 4 and 6 using Clustal Omega and TMHMM v. 2.0 programs to detect cysteines located in or near hydrophobic transmembrane spanning regions, along with identification of palmitoylation potential using CSS-lm 4.0 and Palm-GPS algorithms [61,62,63,64], were performed. Our data indicate that, while coronavirus membrane protein and nsp amino acid sequences vary markedly among human coronaviruses and MHV, their relative positions and number of hydrophobic transmembrane sequences are maintained (Figure 1 and Figure 2).

The coronavirus membrane protein is a type III transmembrane glycoprotein and the most abundant glycoprotein in the virus particle [65]. The membrane protein was shown to coordinate the initial genome packaging within the nucleocapsid and its subsequent integration into the virion membrane with the envelope and spike proteins [66]. Coronavirus membrane protein can fold into either an elongated or compact conformation for which the long form is thought to be important in lipid membrane curvature and virus budding, whereas the compact version likely forms under acidic conditions and during coronavirus entry into the endosome of a new host cell [67]. In silico examination of cysteine residues in SARS-CoV-2 membrane protein predicts palmitoylation only for a similarly positioned cysteine residue in MHV (Figure 1). In silico analysis also uncovered a conserved S(I/F)RL(F/W) motif located within the junction of the third transmembrane sequence and the membrane protein endodomain (Figure 1). Based on homology with the immunoglobulin heavy chain junction region, the S(I/F)RL(F/W) motif may provide conformational flexibility to coronavirus membrane proteins.

In silico examination of nsp3 revealed a conserved cysteine motif C(A)XCXK and palmitoylation target located at the rightward end of its hydrophobic domains for the human coronaviruses examined (Figure 2). Potential nsp4 palmitoylation occurred in the first hydrophobic domain for all the coronavirus families examined except for the SARS-CoV-2 virus (Figure 2). Surprisingly, cysteines detected in the first transmembrane domain of SARS-CoV-1 or other human coronaviruses were replaced with aromatic residues. A BLAST search of GenBank coronavirus taxid (8-27-2020) showed that unlike the reported pangolin intermediary virus isolate MP789 [68], only bat coronavirus isolate RaTG13 was devoid of cysteines and possessed 100% cDNA sequence identity with that of SARS-CoV-2 [69]. Nsp6 contained a conserved -KHKH- junction sequence demarcating the boundaries of the 2nd and 3rd hydrophobic transmembrane domain for all coronaviruses examined, but whose utility during infection has yet to be determined (Figure 2). Although palmitoylation is predicted in seven hydrophobic regions of nsp6, we found no conserved palmitoylation site (Figure 2). While biochemical verification of these predicted palmitoylation sites is warranted, the conserved palmitoylation sites are all located in or near hydrophobic domains, thereby strengthening the argument that SARS-CoV nsp3 and nsp4 membrane interactions require palmitoylation for simple tethering or for more extensive ER reshaping into multilamellar vesicles and DMV pairing.

## 6. Fatty Acid Synthase (FASN) and AMP-Activated Protein Kinase (AMPK): Potential Antiviral Targets to Block SARS-CoV-2 Replication and Virus Assembly

When considering the use of lipid-modifying drugs as potential SARS-CoV-2 antivirals, we defined an antiviral as one that targets specific viral proteins or interferes with a critical step in the virus life-cycle. While sterol-altering drugs such as statins are proven to mitigate inflammation and are currently investigated to lessen the cytokine storm in COVID-19 patients (1 March 2020, clinicaltrials.gov), their use as COVID-19 antivirals remains controversial [70,71]. Evidence suggests that statins may reduce inflammation and interfere with SARS-CoV-2 interaction with the ACE2 receptor. However, statins may also interact with currently used COVID-19 treatments including the glucocorticoid, dexamethasone, or with protease inhibitor antivirals and reduce their potency [72]. Further, it is unclear whether statins may exacerbate COVID-19 pneumonia [73]. We elected not to pursue statins as antivirals seeing that they do not block virus replication for other enveloped RNA viruses, namely hepatitis C virus and influenza virus [74,75,76,77], and we do not expect statins to impact viral protein palmitoylation or the level of fatty acids required during assembly of replication organelles [37]. 

**Figure 3:**
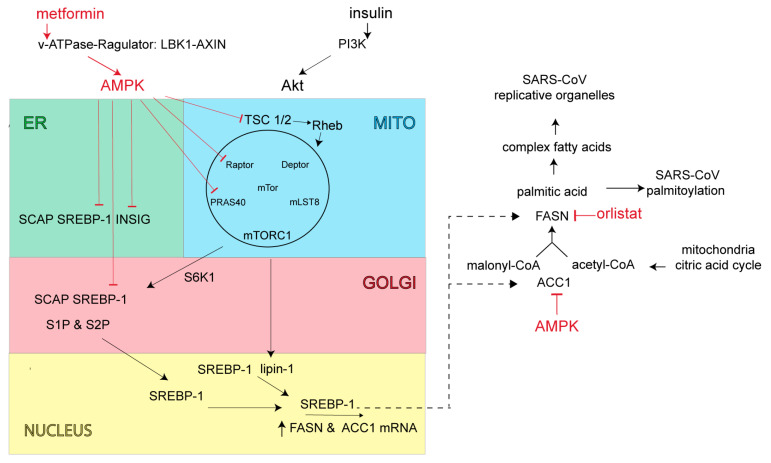
Overview of the lipid synthetic pathway. Insulin activation of mitochondrial (MITO) mTORC1 leads to the phosphorylation of S6K1 and lipin-1. Activated S6K1 acts on ER-resident SREBP-1 resulting in SREBP-1 transport to the Golgi, proteolytic maturation by S1P and S2P, with eventual nuclear entry and transcriptional activation of fatty-acid anabolic genes FASN and ACC1. mTORC1 activation of lipin-1 results in release of perinuclear-bound SREBP-1 for further transcriptional activation of fatty-acid anabolic genes. AMPK blocks fatty acid synthesis by acting directly on the ACC1 enzyme, or indirectly by lowering cellular FASN and ACC1 enzymes through deactivation of mTORC1 or by preventing ER SREBP-1 transport/maturation to/at the Golgi and ultimately preventing lipid gene transcription.

SARS-CoV-2 along with closely related Middle East respiratory syndrome β-coronavirus (MERS-CoV) increase PI3K/AKT/mTOR/S6K signaling activity, which is expected to increase production of lipid anabolic enzymes including FASN and acetyl-CoA carboxylase (ACC1) (Figure 3) [78,79]. FASN is the key cellular enzyme involved in palmitate synthesis [80]. FASN uses acetyl-CoA and malonyl-CoA as substrates to form palmitate, which in turn is used for the palmitoylation of proteins or further processed into more complex lipids for the construction of viral envelopes and replication organelles. FASN inhibitors act primarily through allosteric inhibition of FASN β-ketoacyl reductase activity, resulting in altered lipid membrane synthesis and protein palmitoylation [81]. Acetyl-CoA carboxylase is also a key metabolic enzyme in fatty acid biosynthesis. ACC1 converts mitochondria-derived acetyl-CoA to malonyl-CoA, which in turn serves the dual purpose of directing metabolic intermediates towards fatty acid synthesis or producing malonyl-CoA, and the allosteric inhibitor of carnitine palmitoyl transferase 1 [82]. This latter enzyme acts as the rate-limiting step in fatty acid degradation [82]. Control of fatty acid biosynthesis by inhibiting ACC1 or FASN enzymatic activity is expected to attenuate virus production.

Beyond direct inhibition of FASN and ACC1 enzyme activities, cellular FASN and ACC1 levels are controlled at transcription by the sterol regulatory element-binding protein (SREBP)-1 (Figure 3) [83]. The SREBP-1-dependent lipogenic pathway is normally stimulated by food ingestion, but excessively activated in the setting of obesity-linked insulin resistance [84].

Under conditions of low energy demand and sufficient fatty acid stores, SREBP-1 resides in the ER in an inactive form as a stable complex with the SREBP-cleavage activating protein (SCAP) and the insulin-induced gene protein (INSIG) [85]. INSIG bound to SREBP-1 maintains SREBP-1 in the ER and prevents SREBP-1 transport and proteolytic processing/maturation in the Golgi. During times of insufficient cellular fatty acids, INSIG is ubiquitylated and degraded, allowing SREBP-1-SCAP transport to the Golgi complex, whereby SREBP-1 is proteolytically processed by site-1 and site-2 proteases (S1P, S2P). Upon proteolytic maturation, SREBP-1 is translocated to the nucleus for transcriptional activation of FASN and ACC genes [86]. In cases of T2DM or obesity, where chronic elevation of insulin leads to continued increases in lipid synthesis, mechanistic findings indicate that cellular INSIG levels were reduced [87]. With heightened levels of insulin there is also increased PI3K production of phosphatidylinositol (3,4,5)-triphosphate (PIP3), activation of Akt (AKR mouse thymoma) kinase and increased nuclear SREBP-1 levels via Akt activation of the mammalian target of rapamycin (mTORC1) signaling complex (Figure 3) [88]. mTORC1 is comprised of the mTOR kinase, the adapter protein regulatory-associated protein of mTORC1 (Raptor), the mammalian lethal SEC13 protein 8 (MLST8, also called GβL), as well as its inhibitory subunits, proline-rich Akt substrate-1 (PRAS1) and Akt target domain-interacting protein (DEP) TOR. One of the most important sensors involved in the regulation of mTORC1 activity is the tuberous sclerosis complex (TSC) heterodimer (TSC1 and TSC2). TSC acts as a GTPase for the Ras-related GTPase, Rheb (Ras homolog enriched in brain). As a Rheb-specific GTPase, TSC negatively regulates mTORC1 signaling by converting Rheb into its inactive GDP-bound state. Insulin stimulation and Akt direct phosphorylation inactivates TSC GTPase to promote GTP-Rheb activation and increase mTORC1 signaling activity. Akt also increases mTORC1 activity by phosphorylating PRAS40 (proline rich Akt substrate 40 kD), normally a negative regulator of mTORC1. mTORC1 promotes phosphorylation of lipin-1, a phosphatidic acid phosphatase and S6-kinase activity. The normally dephosphorylated lipin-1 resides in the nucleus and limits “free form” SREBP-1 nuclear levels. Lipin-1 acts by sequestering nuclear SREBP-1 away from its lipid gene responsive elements [89]. Upon lipin-1 phosphorylation, freed SREBP-1 can now target the lipid gene responsive elements. Activated S6-kinase phosphorylates the ER SREBP-1 complex to enhance SREBP-1 Golgi transport and proteolytic maturation [90]. In cases of T2DM or obesity, where chronic insulin elevation leads to increased lipid synthesis and a lipid-rich environment, one would expect the SARS-CoV to more readily form replication organelles and increase virus production.

To counteract the insulin-induced increase in lipid synthesis and to maintain the cell’s proper ATP to fat storage ratio [91], cellular AMPK turns down lipid synthesis directly through phosphorylation of the rate-limiting enzyme ACC1 and indirectly through interruption of mTORC1 signaling and subsequent SREBP-1 processing (Figure 3). AMPK inhibits mTORC1 signaling by phosphorylating TSC2 and converting Rheb into an inactive GDP-bound state, thus switching off Akt stimulation of mTORC1. AMPK also phosphorylates Raptor which leads to 14-3-3 protein binding and mTORC1 inhibition [92]. AMPK also directly blocks the SREBP-1 signaling pathway by phosphorylating both SREBP-1 and INSIG. INSIG phosphorylation blocks INSIG ubiquitination and degradation resulting in SREBP-1 retention in the ER [93]. AMPK phosphorylation of SREBP-1 prevents SREBP-1 proteolytic processing/maturation and ultimately lipid gene transcription [94]. As SREBP-1 controls its own transcription, any increase in INSIG stability or prevention of SREBP-1 maturation would also act as a negative feedback control to further reduce overall SREBP-1 levels and ultimately cause a reduction in lipid enzymes and lipid synthesis [95]. 

A reduction in lipid biosynthesis through AMPK’s actions is expected to attenuate coronavirus replication organelle formation as well as viral protein palmitoylation and virus production (Figure 3). As an added benefit, AMPK’s action on mTORC1 will reduce cellular NFκB activity along with both cellular and systemic inflammatory responses and T-cell activation [96], which should diminish or mitigate aberrant inflammatory responses often seen in severe SARS-CoV-2 infection [97].

## 7. Potential Use of Orlistat and Metformin in Controlling SARS-CoV-2 Protein Palmitoylation and Organelle Assembly 

There are a number of experimental compounds in (pre)clinical testing that inhibit FASN [81] or activate AMPK [98], and could serve as potential anti-SARS-CoV antivirals. However, only FASN inhibitor, orlistat, or AMPK activator, metformin, sold under the brand names Xenical and Glucophage, respectively, are approved for widespread clinical use and have well-established safety profiles. Orlistat is prescribed for treatment of obesity and obesity-related T2DM [99,100,101,102]. Orlistat is a saturated derivative of endogenous lipstatin isolated from *Streptomyces toxytricini*, which covalently binds to the active site of the thioesterase domain of FASN and directly inhibits FASN enzymatic activity (Figure 3) [103,104]. The recommended orlistat dose is one 120 mg capsule taken orally 3× daily during fat-free meals [105]. While the primary site of orlistat accumulation is within the gut, most of the medication (95–97%) is unabsorbed and excreted in feces; however, early clinical studies have detected low but measurable levels of orlistat in the serum when doses were prescribed above the recommended 120 mg [106]. Metformin is a biguanide derivative widely used to treat T2DM [107]. Metformin was shown to be clinically superior in reducing glucose levels with little induction of hypoglycemia or weight gain and to reverse hepatic steatosis, improve insulin sensitivity, and improve atherosclerosis and cardiovascular dysfunction [107]. Metformin’s mechanism of action was once thought to be mitochondrial, involving complex I of the electron transport chain for increased AMP and activation of AMPK [107]. However, more recent findings show that metformin activates the AMPK cascade via the formation of v-ATPase/Ragulator complex in association with liver kinase B1 (LKB1) and axis inhibition protein 1 (AXIN1). Once formed, this larger complex leads to AMPK activation and the switching off of mTORC1 (Figure 3) [108]. While metformin’s actions on AMPK and mTORC1 also lead to changes in glucose and protein metabolism, mitochondrial biogenesis and mitochondrial autophagy that may impact SARS-CoV-2 replication, we focused on metformin’s action on lipid metabolism. Metformin’s action through AMPK reduces lipid storage, increases fatty acid oxidation, inhibits glycolysis and blocks lipogenesis. We expect metformin, acting through AMPK, to turn down palmitate synthesis and virus protein palmitoylation directly through phosphorylation of the rate-limiting enzyme ACC1. We also expect AMPK to reduce overall fatty acid synthesis and replication organelle assembly by interrupting mTORC1 signaling and subsequent SREBP-1 processing (Figure 3). In addition, orlistat’s direct inhibition of FASN enzymatic activity should impair palmitate production and ultimately, viral protein palmitoylation (Figure 3).

### 7.1. Insights from Other Lipid-Dependent Viruses for Use of Orlistat and Metformin in COVID-19 Treatment 

**Figure 4:**
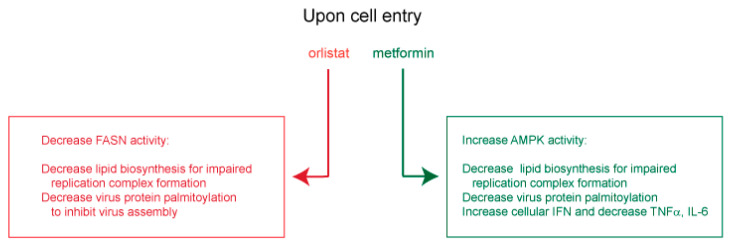
Predicted mechanisms of action used by orlistat and metformin to inhibit SARS-CoV-2 replication.

In other viral settings, orlistat and metformin were shown to inhibit replication of several flaviviruses [109,110,111,112], as well as hepatitis B virus and HCV [113,114,115,116,117], coxsackievirus B3 and varicella-zoster virus [118,119]. For these viruses, orlistat was shown to inhibit lipid-vesicle restructuring, viral genome replication, virion protein palmitoylation [109,110,111,118] and virus entry [113,114]. Metformin was shown to decrease lipid (palmitate) synthesis [119], viral RNA transcription and protein synthesis [115,116], as well as virus-dependent glycolysis, while increasing IFN production and blocking inflammatory cytokines [112,117]. From these observed antiviral mechanisms, orlistat and metformin are expected to decrease FASN and activate AMPK, respectively, for a predicted inhibition of viral replication organelle formation, and to block spike, membrane and envelope palmitoylation and subsequent virus assembly. Orlistat and metformin may also enhance innate cellular immunity by promoting IFN expression and suppressing deleterious IL-6 and TNFα (Figure 4).

### 7.2. Potential Clinical Utility of Orlistat and Metformin in COVID-19 Treatment

Orlistat was shown to decrease IL-6 and TNFα in diabetic patients [120], and to reduce systemic inflammation and pancreatitis-induced death in obese (ob/ob) mice as measured by a lower mortality incidence and decreased serum levels of TNFα, MCP-1 and IL-6 in an acute pancreatitis mouse model [121]. Orlistat was shown to reduce intestinal microsporidiosis caused by *Enterocytozoon* species, as seen by a decrease in fecal spores and spore infectivity [122]. Like the lipid dependency of RNA viruses, orlistat-treated mice exhibited a direct inhibition of microsporidia viability through inhibition of spore-dependent phospholipid synthesis and prevention of parasitophorous vacuoles required during *Enterocytozoon* spore development.

Clinical and animal studies in a variety of disease settings lend strong support for metformin as a SARS-CoV antiviral. Metformin activation of AMPK to enhance IFN signaling, decrease proinflammatory cytokines and restore immune homeostasis could abrogate cytokine storm severity in COVID-19 disease [123,124]. While clinical testing of metformin as a systemic immunometabolic drug is ongoing [125], use of metformin has already demonstrated reduced inflammation in patients and reduced insulitis in mice. Metformin has been shown to inhibit autoreactive and proinflammatory cells and to restore immune homeostasis [126], as well as to reduce IL-6, TNFα and intracellular adhesion molecule-1 (ICAM-1) in women with polycystic ovary syndrome [127], and to lower asthma-related hospitalization and asthma exacerbation in diabetic patients [128]. Metformin’s lowering of asthma events is postulated to act through AMPK and attenuate eosinophilic-driven inflammation [129,130]. Metformin was shown to reduce non-alcoholic fatty liver disease and intestinal inflammation in murine models [131,132]. 

Consideration of metformin use as an anti-SARS-CoV antiviral to alleviate enteric pathology stems from its ability to decrease gut microbiome dysregulation and chronic bowel inflammation in T2DM [133] and inflammatory bowel disease [134]. In addition, metformin decreases the frequency of pathogenic Th17 cells and increases the frequency of beneficial regulatory T cells [134,135] through activation of AMPK and subsequent reduction in activated NFκB [132]. Metformin may reduce SARS-CoV inflammatory signals in infected cells by altering AMPK-dependent signaling and down-stream suppression of cytokine gene activation.

### 7.3. Therapeutic Application of Orlistat and Metformin 

The optimal daily dose recommended for metformin is 2000 mg. When taken orally and during meals, like orlistat, the primary site of accumulation is within the gut, with elimination in the urine and feces [107]. A review of potential drug toxicity indicates that orlistat is well-tolerated; however, COVID-19 patients may need to be monitored for rare occurrence of orlistat-induced hepatic injury [136,137]. In rare cases (4.3/100,000), metformin may decrease lactate uptake by the liver, thereby increasing the risk of lactic acidosis. Its use in patients with severe renal insufficiency, acidosis, congestive heart failure, liver disease and hypoxemia should be monitored [138]. 

It has been suggested that metformin use during COVID-19 treatment may increase SARS-CoV infectivity by augmenting the level of cellular ACE2 (by approximately 0.8-fold) [139,140]. This increase may outweigh the benefits of preventing SARS-CoV-induced lung inflammation and damage [141,142] or, in our view, metformin’s inhibition of lipid synthesis and virus replication. In added support for metformin use to treat COVID-19, we note that SARS-CoV-1 spike protein employs the metzincin protease family member TNF-α-converting enzyme (TACE) to cleave ACE2, which then enables virus entry [143,144,145]. Prevention of TACE protease activity attenuates SARS-CoV infection [144]. Since metformin can inhibit other metzincin family members, namely metalloproteinase (MMP)-2 and MMP-9 [146], one might infer that metformin acts in a similar inhibitory fashion on TACE protease to prevent ACE2 cleavage and virus entry. The higher levels of cellular ACE2 seen following metformin treatment may in fact reflect uncut and dysfunctional ACE2 SARS-CoV receptors. 

Potential drug interactions with currently proposed COVID-19 treatment modalities can easily be avoided with simple dietary changes, increased dosage or offsets in time of drug administration (i.e., fat-soluble vitamins, cyclosporine A, https://clinicaltrials.gov, search term “COVID-19”, 1 March 2020). 

## 8. Conclusions and Future Directions 

**Figure 5:**
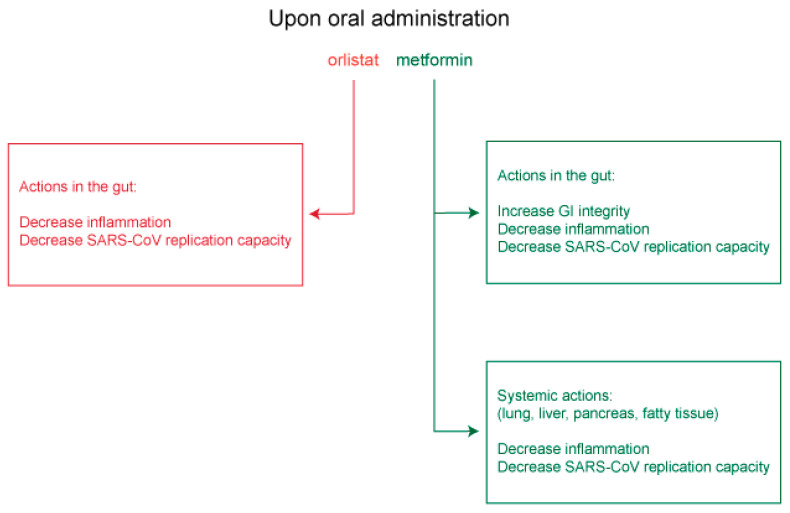
Predicted systemic actions by orlistat and metformin in inhibiting COVID-19 disease and pathologies.

Approved medicines such as orlistat and metformin that act respectively on FASN and AMPK, and which are expected to inhibit virus replication and assembly or to promote gastrointestinal integrity and decrease deleterious systemic inflammation, merit serious consideration for clinical testing as anti-COVID-19 therapies (Figure 5). 

In support for our hypothesis that metformin may be useful in COVID-19 management, work by Zhu et al. (2020) indicates that individuals using drugs to control their T2DM diabetes faired markedly better in hospitalization outcomes compared to those who did not use drugs [147]. Upon further examination of the authors’ data (Zhu et al., Table 2), we noted that COVID-19 patients using metformin faired best overall (27.3%) [147]. Although the small sample size did not allow for statistical significance using Z-test for proportions, the data trend did show that metformin use improved hospitalization outcomes more than (>) the anti-T2DM alpha-glucosidase inhibitors (26.7%), > thiazolidinediones (22.2%), > sulfonylurea (20.1%), > meglitinides (20.0%), > dipeptidyl peptidase-4 inhibitors (20.0%) and > insulin (12.1%). Subsequent studies examining metformin use in diabetic individuals hospitalized for COVID-19 infection showed an association with a lower risk of death [148,149].

Clinical studies for orlistat use and COVID-19 outcomes await further investigation. However, a yet to be published review of orlistat effectiveness in lowering HCV viral load in the phase 4 clinical study (NCT00207311, clinical trials.gov) may give clearer medical guidance and a possible clinical path forward to effectively treat SARS-CoV-2 infection using orlistat. 

If used in conjunction with other antivirals and anti-inflammatory drugs, orlistat and/or metformin may further suppress SARS-CoV-2 replication through overall lowering of lipid synthesis for a reduction in replication organelles and prevention of viral protein palmitoylation, thereby lowering immune-induced morbidity in high-risk and affected patients. One may envisage clinical prophylaxis using orlistat or metformin in patients following a positive test for the disease and at an early stage in their disease progression. If incorporated judiciously with other COVID-19 antivirals, both drugs may act synergistically with these antivirals to hasten patient recovery before the need for more invasive intensive care management.
